# Clinically applicable CD34^+^-derived blood dendritic cell subsets exhibit key subset-specific features and potently boost anti-tumor T and NK cell responses

**DOI:** 10.1007/s00262-021-02899-3

**Published:** 2021-04-01

**Authors:** Jesper van Eck van der Sluijs, Diede van Ens, Soley Thordardottir, Denise Vodegel, Inge Hermens, Anniek B. van der Waart, J. H. Frederik Falkenburg, Michel G. D. Kester, Iris de Rink, Mirjam H. M. Heemskerk, Jannie Borst, Nicolaas P. M. Schaap, Joop H. Jansen, Yanling Xiao, Harry Dolstra, Willemijn Hobo

**Affiliations:** 1 Department of Laboratory Medicine, Laboratory of Hematology, Radboud Institute for Molecular Life Sciences, Radboud University Medical Center, Geert Grooteplein 8, P.O. Box 9101, 6500 HB Nijmegen, The Netherlands; 2 Department of Hematology, Leiden University Medical Center, Leiden, The Netherlands; 3 Genomics Core Facility, The Netherlands Cancer Institute, Amsterdam, The Netherlands; 4 Department of Immunology and Oncode Institute, Leiden University Medical Center, Leiden, The Netherlands; 5 Department of Hematology, Radboud University Medical Center, Nijmegen, The Netherlands

**Keywords:** CD34^+^ hematopoietic progenitor cells, Clinical application, Dendritic cells, NK cells, T cells, Vaccination

## Abstract

Allogeneic stem cell transplantation (alloSCT), following induction chemotherapy, can be curative for hemato-oncology patients due to powerful graft-versus-tumor immunity. However, disease recurrence remains the major cause of treatment failure, emphasizing the need for potent adjuvant immunotherapy. In this regard, dendritic cell (DC) vaccination is highly attractive, as DCs are the key orchestrators of innate and adaptive immunity. Natural DC subsets are postulated to be more powerful compared with monocyte-derived DCs, due to their unique functional properties and cross-talk capacity. Yet, obtaining sufficient numbers of natural DCs, particularly type 1 conventional DCs (cDC1s), is challenging due to low frequencies in human blood. We developed a clinically applicable culture protocol using donor-derived G-CSF mobilized CD34^+^ hematopoietic progenitor cells (HPCs) for simultaneous generation of high numbers of cDC1s, cDC2s and plasmacytoid DCs (pDCs). Transcriptomic analyses demonstrated that these ex vivo-generated DCs highly resemble their in vivo blood counterparts. In more detail, we demonstrated that the CD141^+^CLEG9A^+^ cDC1 subset exhibited key features of in vivo cDC1s, reflected by high expression of co-stimulatory molecules and release of IL-12p70 and TNF-*α*. Furthermore, cDC1s efficiently primed alloreactive T cells, potently cross-presented long-peptides and boosted expansion of minor histocompatibility antigen-experienced T cells. Moreover, they strongly enhanced NK cell activation, degranulation and anti-leukemic reactivity. Together, we developed a robust culture protocol to generate highly functional blood DC subsets for in vivo application as tailored adjuvant immunotherapy to boost innate and adaptive anti-tumor immunity in alloSCT patients.

**Supplementary Information:**

The online version contains supplementary material available at 10.1007/s00262-021-02899-3.

## Introduction

Allogeneic stem cell transplantation (alloSCT), following induction chemotherapy, can be curative for hemato-oncology patients [1]. The therapeutic graft-versus-tumor (GVT) effect is mediated by donor-derived CD8^+^ T cells recognizing tumor-associated antigens (TAAs), neo-antigens or minor histocompatibility antigens (MiHAs) on recipient tumor cells [2]. Additionally, donor-derived natural killer (NK) cells play an important role in GVT immunity [3], especially upon KIR-ligand mismatched alloSCTs [4]. However, many patients exhibit suboptimal activation, expansion and/or functionality of tumor-reactive T and NK cells, which contributes to relapse. This underscores the medical need for adjuvant immunotherapy. In this regard, dendritic cell (DC) vaccination is highly attractive as DCs are the key orchestrators of innate and adaptive immunity. The last decades, ex vivo-generated monocyte-derived DC vaccination was shown to be feasible and well-tolerated in cancer patients. However, clinical responses were limited to only few patients [5]. To increase the potency and therapeutic efficacy of DC vaccination, alternative strategies are actively pursued, including the use of natural human DC subsets due to their unique functional properties and cross-talk capacity [6–8].

Natural human DCs can be divided in two major subsets: conventional DCs (cDCs) and plasmacytoid DCs (pDCs). cDCs can be further subdivided in type 1 cDCs (cDC1s; CD141^+^CLEC9A^+^) and type 2 cDCs (cDC2s; CD1c^+^) [9]. These cDCs confer immunity to infection and produce the pro-inflammatory cytokine IL-12p70, which promotes T helper 1 and cytotoxic T lymphocyte (CTL) responses. On the other hand, pDCs are key players in anti-viral immunity and produce high levels of type I interferons (IFNs), promoting innate and adaptive immunity [10]. Recently, the feasibility and safety of peripheral blood (PB)-derived pDC and cDC2 vaccination was demonstrated in melanoma patients and induction of anti-tumor CD8^+^ T cell responses was observed [11, 12]. In addition, cDC1s are highly attractive for DC-based immunotherapy because of their superior capacity to cross-present exogenous tumor antigens to CD8^+^ T cells, and activation of NK cells [13–15]. Yet, cDC1s have not been clinically evaluated so far, which can be attributed to their low frequencies in blood (< 0.05% of mononuclear cells), making it difficult to obtain sufficient numbers for vaccination [9]. Hence, we and others established ex vivo culture protocols to generate natural DC subsets from human CD34^+^ hematopoietic progenitor cells (HPCs) [16–21]. Most of these methods rely on expansion of cord blood or bone marrow-derived CD34^+^ HPCs on OP9 murine bone marrow-derived stromal cells retrovirally transduced with Notch ligands. In contrast, we developed an alternative good manufacturing practice (GMP)-compliant protocol devoid of animal components, allowing easier translation to the clinic. Our protocol uses a small fraction (≤ 5%) of the G-CSF mobilized CD34^+^ HPCs from the donor stem cell graft to provide tailored adjuvant immunotherapy post alloSCT. 

Here, we describe for the first time a clinically applicable ex vivo culture protocol that enables simultaneous and reproducible generation of high numbers of pDCs, cDC2s and the rare cDC1s from G-CSF mobilized CD34^+^ HPCs, derived from donor stem cell grafts. These ex vivo-generated DC subsets approximate their blood counterparts on transcriptomic level. In more detail, we demonstrated that the ex vivo-generated cDC1s harbor the distinctive phenotypic and functional features of their in vivo blood counterpart, including IL-12p70 and TNF-α production, superior antigen-cross presentation and potent induction of MiHA-specific T cell functionality and leukemia-reactive NK cell responses. Together, these ex vivo-generated DC subsets hold strong potential as adjuvant immunotherapy to boost innate and adaptive anti-tumor immunity and improve relapse-free survival of alloSCT patients.

## Materials and methods

### Patient and donor material

CD34^+^ HPCs and T cells were obtained from leukapheresis material of G-CSF-mobilized donors. For MiHA-specific T cell expansion assays, cryopreserved peripheral blood mononuclear cells (PBMCs) containing HA-1 or LRH-1 CD8^+^ memory T cells obtained from alloSCT patients were used. Healthy donor PBMCs were isolated from buffy coats obtained from Sanquin Blood Supply Foundation. For primary AML killing assays, cryopreserved bone marrow (BM) samples, containing ≥ 90% blasts, of AML patients obtained at diagnosis were used. Patient characteristics are described in Table S1. Cellular material was obtained in accordance with the Declaration of Helsinki and institutional guidelines and regulations (CMO 2013/064).

### Ex vivo***-***generation of DCs from G-CSF mobilized CD34^+^ HPCs

**Fig. 1:**
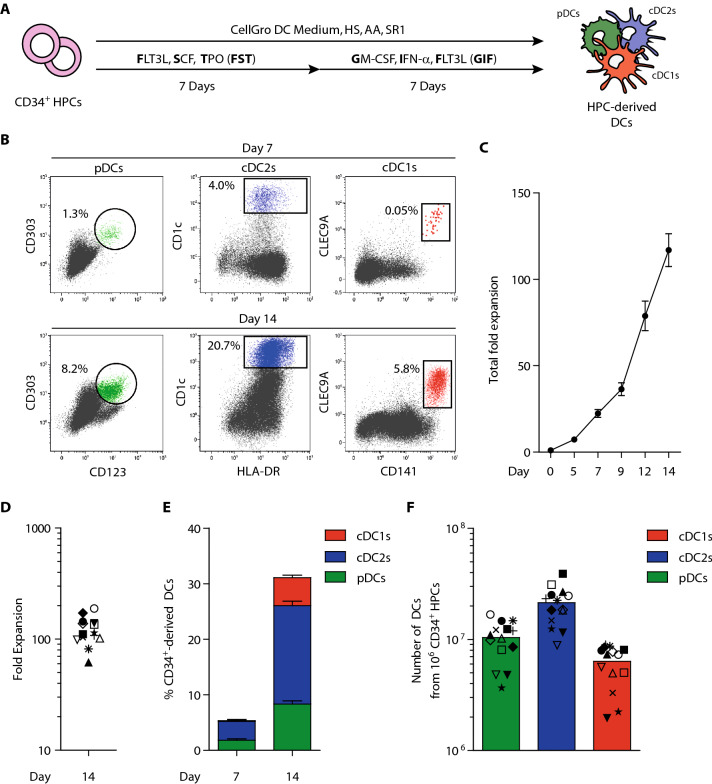
Simultaneous generation of pDCs, cDC2s and cDC1s from CD34^+^ HPCs using a single clinically translatable culture protocol. **a** Schematic overview of the culture protocol. **b** Representative dot plots showing ex vivo-generated pDCs (green), cDC2s (blue) and cDC1s (red) within total cultured cells. Gating strategy is shown in Fig S1. **c** Fold expansion of total cultured cells over time. Data is shown as mean ± SEM (*n* = 15). **d** Fold expansion of total cultured cells at day 14. Each symbol represents an independent CD34^+^ HPC donor (*n* = 14). **e** Frequencies of ex vivo-generated pDCs (green), cDC2s (blue) and cDC1s (red) at day 7 and day 14 of culture. Data is shown as mean ± SEM (*n* = 14). **f** Absolute numbers of ex vivo-generated pDCs (green), cDC2s (blue) and cDC1s (red) from 1 × 10^6^ CD34^+^ HPCs. Each symbol represents an independent CD34^+^ HPC donor (corresponding with symbols in figure D) with the bar representing the mean value (*n* = 14). pDC, plasmacytoid dendritic cell; cDC2s, conventional type 2 dendritic cell; cDC1, conventional type 1 dendritic cell; G-CSF, granulocyte-colony stimulating factor; HPCs, hematopoietic progenitor cells; SEM, standard error of the mean

CD34^+^HPCs were isolated from G-CSF mobilized blood using CD34 MicroBeads (Miltenyi Biotec) and purity (> 90%) was checked using flow cytometry. G-CSF mobilized CD34^+^HPCs were cultured for 14 days in CellGenix® GMP DC medium (CellGenix) with 2% HS, 1 µM StemRegenin1 (SR1, Cellagen Technology) and 50 µg/mL ascorbic acid (AA, Sigma Aldrich). From day 0–7, medium was supplemented with ‘FST’ cocktail: 100 ng/mL recombinant human (rh) FLT3L, 100 ng/mL rhSCF and 100 ng/mL rhTPO (Immunotools). On day 7, cells were harvested, washed and resuspended in medium supplemented with ‘GIF’ cocktail: 800 IU/mL rhGM-CSF (Immunotools), 1000 IU/mL rhIFN-α (Roche) and 100 ng/mL rhFLT3L, and cultured till day 14 (Fig. 1a). Ex vivo-generated and/or in vivo (PB-derived) DC subsets were sorted using the Aria SORP sorter (BD Biosciences) and all functional assays were performed using freshly sorted cells (Fig S2).

### DC maturation and cytokine release

Enriched DCs were matured and cytokine release was investigated according to previously reported procedures, with the following alteration: pDCs were matured using 0.5 µM CpG-P (Miltenyi Biotec). No rhIL-4 was added as survival factor to cDCs [18].

### Flow cytometry

Extracellular and intracellular flow cytometry stainings were performed as described previously [12, 22]. Antibodies are listed in Table S2.

### RNA deep sequencing

Ex vivo DC subsets were generated from 3 individual CD34^+^ donors and in vivo DC subsets were derived from 3 different healthy donor buffy coats. Sorted cell pellets (0.2–0.4 × 10^6^ ex vivo-generated or 0.8–4 × 10^4^ in vivo DC subsets) were dissolved in Trizol reagent (Ambion life technologies) or RLT lysis buffer (Qiagen) and delivered to the Netherlands Cancer Institute Genomics Core Facility. Here, mRNA was isolated and deep sequencing was performed using a HiSeq200 (Illumnia). Sequence reads, single end base pairs, were aligned with TopHat software to the Ensemble gene set *Homo sapiens* GRch37.66. Reads were counted using htseq-count software. Strandedness of the reads was taken into account. Hierarchical clustering was performed in R and 1-correlation was used as distance function. Reads for protein-coding genes were normalized and analyzed for differential expression using R package limma/edgeR.

### Migration assay

In vitro migration potential of ex vivo-generated cDCs was assessed using the 5-µM pore transwell system (Corning™). 1 × 10^5^ mature cDCs were loaded in the inserts in IMDM/10% FCS. Next, IMDM/10% FCS with/without 250 ng/mL CCL21 (Immunotools) was added to the lower compartment. cDCs were cultured for 3 h at 37 °C, whereupon the number of migrated cells in the lower compartment was analyzed using flow cytometry. As positive control, 1 × 10^5^ cDCs were directly transferred to the lower compartment. Percentage of migrated cells was calculated as follows: (number of migrated cells in CCL21 condition/number of cells in positive control)*100%.

### Allogeneic mixed leukocyte reaction and cytokine release

Ex vivo-generated cDCs were seeded at 1.25–5 × 10^3^ cells per well in round-bottom 96-wells plates in IMDM/10% FCS/PS and matured overnight. The following day, CD3^+^ T cells were isolated from healthy donor PBMCs using MACS Pan T cell Kit (Miltenyi Biotec) and labeled with 1.25 µM carboxylfluorescein succinimidyl ester (CFSE) cell proliferation dye (Thermo Fisher). 5 × 10^4^ CFSE-labeled T cells were added to the cDCs and subsequently co-cultured at 37 °C, 5% CO_2_ (cDC:T cell ratio 1:40 and 1:10). After 5 days, supernatant was harvested and T cell proliferation was assessed using flow cytometry. IFN-*γ* release was analyzed by ELISA (Standard, Bender MedSystem, Coating Antibody, Endogen, Biotinylated Detection Antibody, Mabtech).

### Antigen-specific T cell assays

Antigen-specific T cell assays were performed as described before [18], with the following alterations. In brief, for cytomegalovirus (CMV) assays, ex vivo cDCs were generated from HLA-A2^+^ and/or -B7^+^ HPC donors having CMV-reactive CD8^+^ T cells. For HA-1 and LRH-1 assays, cDCs were generated from HLA-A2^+^HA-1^−^ or HLA-B7^+^LRH-1^−^ HPC donors. Mature cDCs were loaded CMV short-peptide (RPH: RPHERWGFTVL or TPR: TPRVTGGGAM), HA-1 short-peptide (VLHDDLLEA) or LRH-1 short-peptide (TPNQRQNVC; LUMC-IHB peptide facility). Fold expansion was calculated based flow cytometric percentages and absolute numbers of cells.

### Cross-presentation assay

First HA-1 transgenic T cell receptor (TCR) transduced CD8^+^ T cells were used as a model system. Here, 1 × 10^4^ ex vivo cDCs, generated from HLA-A2^+^HA-1^−^ HPC donors, were pre-incubated with/without 2.5 µg/mL cytochalasin D (CytD, Sigma Aldrich) for 1 h at 37 °C in 75 µL plain IMDM in round-bottom 96-wells plates. Then, 25 µL IMDM with/without HA-1 short-peptide or HA-1 long-peptide (VARFAEGLEKLKECVLHDDLLEARRPRAHEZL) was added (final peptide concentration 5 µM) and cells were incubated for 2 h at 37 °C. Whereupon overnight maturation was induced as described before. Next, 100µL supernatant was harvested and 100µL IMDM/10% FCS containing overnight rested 1 × 10^4^ HA-1 transgenic TCR T cells were added [23] (cDC:T cell ratio 1:1), CD107a antibody (Biolegend) and Brefeldin A (BD) was added to each well. The following day, T cell activation and intracellular cytokine production was analyzed using flow cytometry. Next, alloSCT patient PBMC containing low frequencies of HA-1 specific CD8^+^ T cells were used. Here, 1 × 10^5^ ex vivo cDC1s, generated from HLA-A2^+^HA-1^−^ HPC donors, were co-cultured with 1 × 10^6^ patient-derived PBMCs in IMDM/10% HS. At day 4, 1 mL fresh IMDM/10% HS containing 50 U/mL rh IL-2 (Immunotools) and 5 ng/mL rh IL-15 (Immunotools) was added. At day 7, cells were harvested and HA-1 specific T cell expansion was assessed using flow cytometry.

#### NK cell cytotoxicity assay

NK cell cytotoxicity assays were performed as described previously [18], with the following alterations: 1 × 10^3^, 1 × 10^4^ or 5 × 10^4^ DCs were seeded and matured for 1 h, followed by TLR-ligand washout. Furthermore, 4-h (cell line) or 48 h (primary AML cells) degranulation and cytotoxicity assays were performed using 1.25 µM CFSE-labeled target cells (THP-1, KG1a, HL-60 and primary AML) in NK:Target at ratio 2:1.

#### Statistical analyses

Statistical analyses were performed using GraphPad Prism 5.03. Statistical differences were determined as indicated in graph legend. *P* values < 0.05 were considered statistically significant.

## Results

### Ex vivo pDCs, cDC2s and cDC1s are simultaneously generated from G-CSF mobilized CD34^+^ HPCs and approximate their natural counterparts

Here, we established a clinically applicable ex vivo protocol for simultaneous large-scale generation of pDCs, cDC2s and the rare cDC1s from human donor-derived G-CSF mobilized CD34^+^ HPCs (Fig. 1a). These CD34^+^ HPC-derived DC subsets were identified within total cultured cells using conventional markers: CD123^+^CD303^+^ pDCs, HLA-DR^+^CD1c^+^ cDC2s and CD141^+^CLEC9A^+^ cDC1s (Fig. 1b, Fig S1). On average, cells expanded 110-fold (range 56–188 fold) during the culture process (Fig. 1c–d). At day 7, low frequencies of pDCs and cDC2s could be detected. Notably, at day 14, all three DC subsets were present. Together they accounted for 31% of total cultured cells: 8.3% pDCs (range 4.3–11.4%), 17.7% cDC2s (range 13.1–23.1%) and 5.0% cDC1s (range 2.8–8.0%) (Fig. 1e). Starting with 1 × 10^6^ HPCs, we generated 10.4 × 10^6^ (range 3.7–16.9 × 10^6^) pDCs, 21.3 × 10^6^ (range 8.9–39.5 × 10^6^) cDC2s and 6.3 × 10^6^ (range 2.0–9.0 × 10^6^) cDC1s (Fig. 1f).

**Fig. 2:**
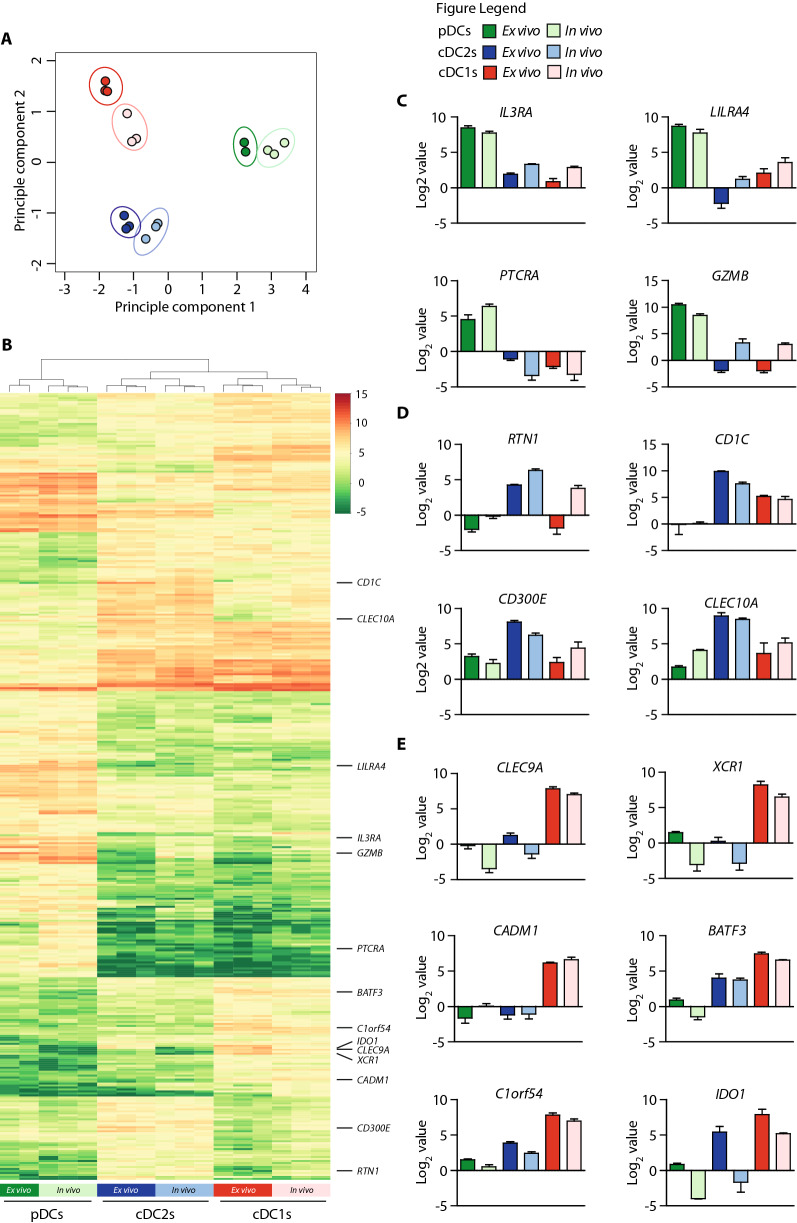
mRNA deep sequencing analyses reveals strong homology of ex vivo-generated DC subsets with their in vivo counterparts. The ex vivo-generated DC subsets vs. in vivo DC subsets were generated/isolated from unrelated donors. **a** Principal component analyses based on 820 differentially expressed protein-coding genes at *p* < 0.05 and Log value > 2 or < -2. **b** Hierarchical clustering and heat map depicting 820 differentially expressed protein-coding genes between pDCs, cDC2s and cDC1s from individual samples according to limma/edgeR (*p* < 0.05 and log_2_ value > 2 or < -2). **c–e** Expression of pDC-related genes (**c**), expression of cDC2-related genes (**d**) and expression of cDC1-related genes (**e**). For ex vivo-generated pDCs *n* = 2, for other DC subsets *n* = 3

Next, we examined to which extent our ex vivo-generated DC subsets resembled the natural blood isolated DC subsets using mRNA deep sequencing. In vivo and ex vivo*-*generated pDCs, cDC2s and cDC1s were sorted based on discriminative marker expression (Fig S2) [24]. 820 protein-coding genes were differentially expressed between all 6 populations at *p* < 0.05 and Log_2_ value > 2 or < -2. Principal component analysis based on these 820 genes separated pDCs from cDCs and cDC2s from cDC1s. Importantly, ex vivo-generated DC subsets clustered closely together with their respective natural counterparts (Fig. 2a). Hierarchical clustering and heatmap analyses using these differentially expressed genes revealed a high similarity between ex vivo-generated DCs subsets and their corresponding in vivo counterparts (Fig. 2b). Accordingly, subset-specific key genes were found to be highly expressed by our ex vivo-generated DC subsets [25]. For instance, pDC-related genes, including *IL3RA*, *LILRA4*, *PTCRA* and *GZMB*, were predominantly expressed in both ex vivo-generated and in vivo pDCs (Fig. 2c). Furthermore, expression of cDC2-related genes, including *RTN1*, *CD1C*, *CD300E* and *CLEC10A* was more pronounced in both ex vivo-generated and in vivo cDC2s compared to pDCs and cDC1s (Fig. 2d). Importantly, cDC1-restricted key genes *CLEC9A*, *XCR1* and *CADM1* were solely expressed on ex vivo-generated and in vivo cDC1s, while cDC1-related genes *BATF3*, *C1orf54* and *IDO1* were higher expressed by both ex vivo-generated and in vivo cDC1s compared to pDCs and cDC2s (Fig. 2e). Additionally, our ex vivo-generated cDC1s, cDC2s and pDCs showed similar *transcription factor* and *toll like receptor* (TLR) expression profiles as their respective in vivo counterparts (Fig. S3a). In conclusion, our novel clinically applicable ex vivo culture protocol allows simultaneous generation of large numbers of all three DC subsets, including the rare cDC1s, which closely match their in vivo counterparts at the transcriptomic level.

### Ex vivo-generated cDC1s phenotypically and functionally resemble the in vivo counterpart

**Fig. 3:**
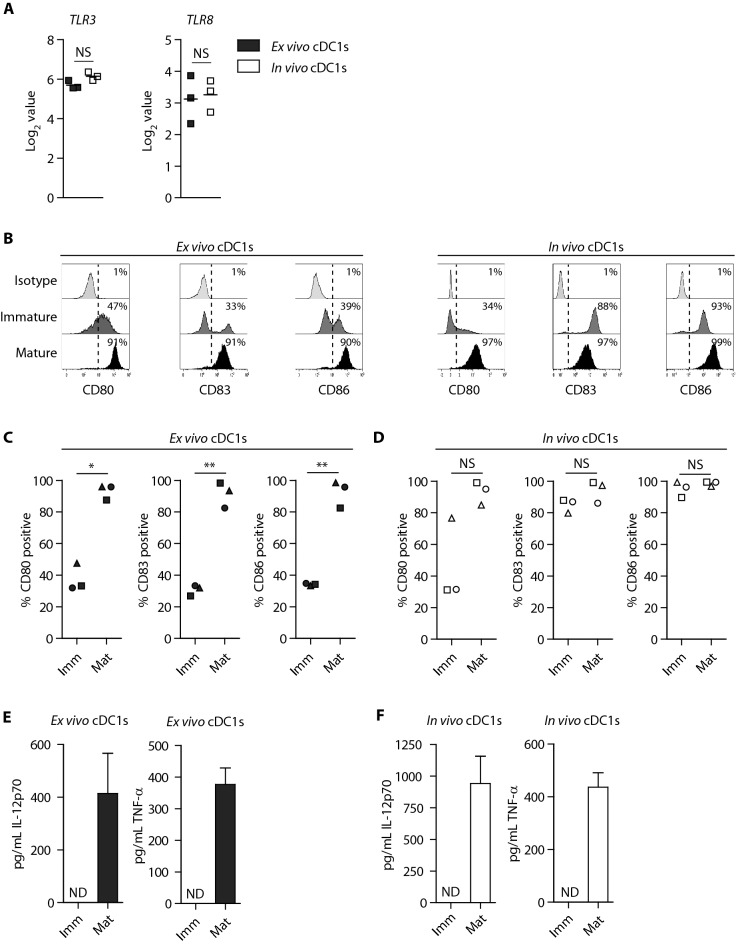
Ex vivo-generated cDC1s exhibit functional resemblance with their in vivo counterpart. The ex vivo-generated DC subsets vs. the in vivo DC subsets were generated/isolated from unrelated donors. **a** Expression of *TLR3* and *TLR8* on ex vivo-generated- and in vivo cDC1s. **b–d** Representative histograms (**b**) and bar graphs **(c–d)** depicting expression of co-stimulatory molecules CD80, CD83 and CD86 on immature and mature versus isotype staining (on immature cDC1s) on ex vivo-generated (**b–c**) and in vivo (**b**, **d**) cDC1s. Lines indicate mean value (*n* = 3). Each symbol represents an independent donor. **e–f** Release of pro-inflammatory cytokines IL-12p70 and TNF-α by immature and mature ex vivo-generated (**e**) or in vivo cDC1s (**f**). Data is shown as mean ± SEM of 6 (ex vivo-generated cDC1s) or 3 (in vivo cDC1s) independent donors. Statistical analysis was performed using paired T test (**c**, **d**). **P < 0.01, *P < 0.05). TLR, Toll like receptor; NS, non-significant; ND, non-detectable; SEM, standard error of the mean

Due to the novelty of large-scale ex vivo-generation of the rare cDC1s for adjuvant immunotherapy post alloSCT, we further characterized this subset in more detail (pDC and cDC2 characterization and cDC2 functional data are provided in supplemental material). First, we demonstrated that both ex vivo-generated and in vivo cDC1s expressed *TLR-3* and *TLR-8* (Fig. 3a, S3a) [9]. Upon TLR3/7/8-mediated maturation, both ex vivo-generated and in vivo cDC1s strongly upregulated expression of CD80 and CD86, while CD83 was only elevated on ex vivo-generated cDC1s (Fig. 3b–d). Furthermore, mature ex vivo-generated and in vivo cDC1s efficiently produced high levels of IL-12p70 and TNF-*α* (Fig. 3e–f). Similarly, ex vivo-generated cDC2s and pDCs highly upregulated the expression of CD80, CD83 and CD86, and produced IL-12p70 (cDC2s) or IFN-*α* (pDCs) following maturation (Fig. S3b–g). Together, these data demonstrate that ex vivo-generated cDC1s share key phenotypic and functional characteristics with the in vivo cDC1 counterpart.

### Ex vivo-generated cDC1s exhibit lymph node homing potential and efficiently boost T cell responses

**Fig. 4:**
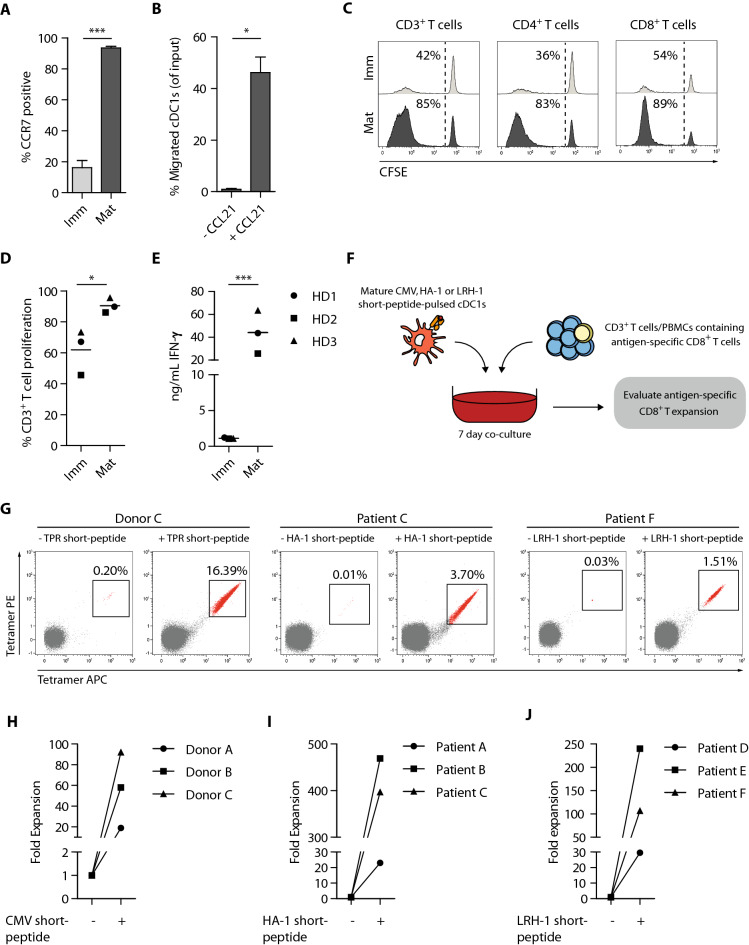
Ex vivo-generated cDC1s possess lymph node homing capacity, efficiently induce alloreactive T cell proliferation and boost expansion of tumor-reactive T cells. **a** Expression of lymph node homing chemokine receptor CCR7 on immature and mature ex vivo-generated cDC1s. Data is shown as mean ± SEM (*n* = 3). **b** Percentage of ex vivo-generated cDC1s migrated to LN homing chemokine CCL21 (250 ng/mL). Data is shown as mean ± SEM (*n* = 3). **c**–**d** Representative histograms (**c**) and bar graph (**d**) showing allogeneic T cells proliferation at day 5 of culture (cDC1:T cell ratio of 1:10). Lines indicate mean value (*n* = 3). **e** Release of IFN-*γ* upon 5 days T cell stimulation in alloMLRs using a cDC1:T cell ratio of 1:10. Line indicates mean value (*n* = 3). **f** Schematic overview of antigen-specific T cell assays. Mature ex vivo-generated cDC1s pulsed with CMV, HA-1 or LRH-1 short-peptide were co-cultured with autologous CD3^+^ T cells (CMV assay) or allogeneic patient-derived PBMCs (HA-1 and LRH-1 assay) for 7 days, whereupon antigen-specific CD8^+^ T cell proliferation was assessed using tetramer-based flow cytometry. **g**–**j** Representative dot plots in which numbers indicate frequencies of TPR (CMV), HA-1 or LRH-1 specific CD8^+^ T cells upon stimulation with ex vivo-generated cDC1s with/without peptide-pulsing (**g)** and graphs showing absolute numbers of CMV, HA-1 or LRH-1 specific CD8^+^ T cells upon stimulation with/without peptide-pulsed ex vivo-generated cDC1s (**h**–**j**). Patient characteristics are shown in supplementary table S1. Statistical analysis was performed using an paired T test (**a**–**b, d**–**e**). ***P < 0.001, *P < 0.05. LN, lymph node; AlloMLRs, allogeneic mixed leukocyte reactions; SEM, standard error of the mean

As effective priming and activation of T cells requires DC homing to lymphoid tissues, we evaluated the expression of lymph node homing molecule CCR7 and corresponding in vitro migration capacity of our ex vivo-generated cDC1s. CCR7 was highly upregulated by cDC1s upon maturation (Fig. 4a), which facilitated efficient in vitro migration of cDC1s toward a CCL21 chemokine gradient (Fig. 4b). Notably, similar results were obtained with ex vivo-generated cDC2s (Fig. S4a–b).

Next, we investigated their T cell stimulatory capacity in allogeneic mixed leukocyte reactions. Notably, mature cDC1s were superior in priming alloreactive T cells, compared to immature cDC1s, as reflected by 90% T cell proliferation and high IFN-γ production (Fig. 4c–e). At a lower cDC1:T cell ratio (1:40), their alloreactive T cell proliferation capacity remained high with 82% of T cells proliferating, though IFN-γ production was ninefold lower (Fig. S4c–d). Importantly, our ex vivo*-*generated cDC1s were equally efficient in priming alloreactive T cells as ex vivo-generated cDC2s (Fig. S4e). 

Additionally, we investigated the potential of our ex vivo-generated cDC1s to induce a recall response of viral- or MiHA-specific CTLs. Hereto, autologous G-CSF-mobilized PB-derived CD3^+^ T cells or patient-derived PBMCs were co-cultured with short-peptide-pulsed cDC1s, whereupon antigen-specific CD8^+^ T cell recall was assessed (Fig. 4f). cDC1s efficiently augmented the expansion of CMV-reactive CD8^+^ T cells, reflected by enhanced frequencies and fold expansion of CMV-specific T cells (Fig. 4g–h). More importantly, ex vivo stimulation with HA-1- or LRH-1 short-peptide-pulsed cDC1s, resulted in enhanced frequencies and expansion of HA-1- and LRH-1-specific CD8^+^ memory T cells (HA-1: 23-fold, 469-fold, 397-fold; LRH-1: 29-fold, 245-fold and 106-fold, respectively) in six patients post alloSCT (Fig. 4i–j). Notably, no antigen-specific T cell expansion was observed in the absence of short-peptide presentation (Fig. 4g–j). Ex vivo-generated cDC2s potently induced antigen-specific T cell expansion, though cDC1s were potentially more powerful as reflected by higher fold expansions (Fig. S4f–h). To summarize, these findings show that ex vivo-generated cDC1s are highly potent in priming alloreactive T cell responses and boosting the expansion of antigen-experienced CMV- and MiHA-specific CD8^+^ memory T cells.

### Ex vivo-generated cDC1s cross-present tumor antigens and activate tumor-reactive T cells

**Fig. 5:**
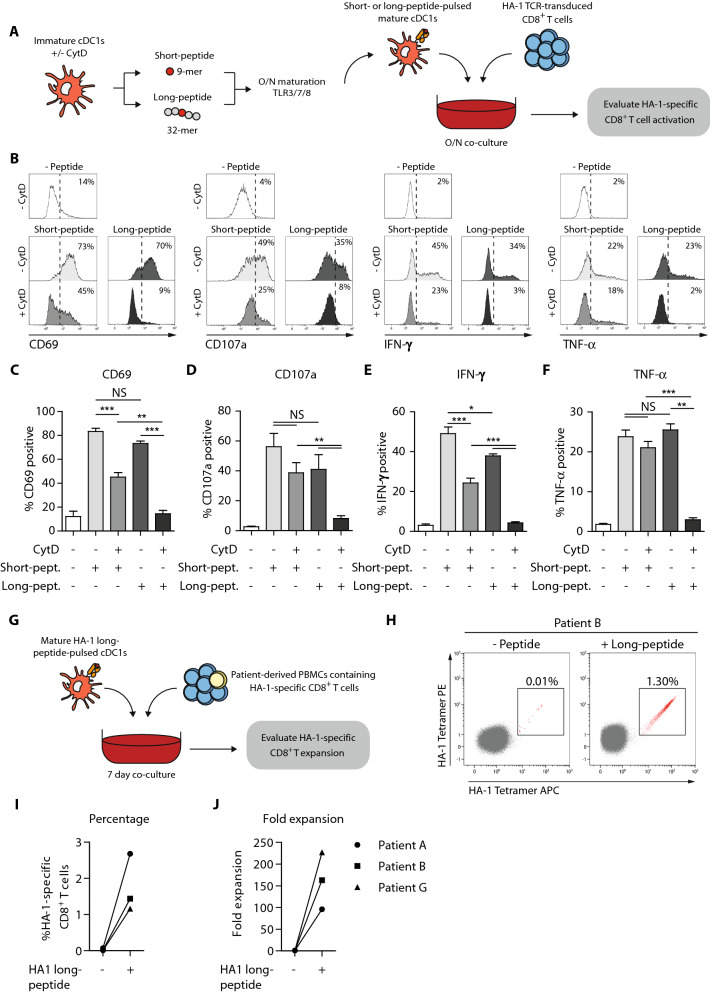
Ex vivo-generated cDC1s superiorly cross-present tumor antigens and activate HA-1-reactive T cells. **a** Schematic overview of antigen cross-presentation assay with HA-1 TCR-transduced T cells. Immature ex vivo-generated cDC1s and cDC2s were treated with/without CytD for 1 h, followed by 2 h incubation with either short- or long HA-1 peptide, whereupon overnight maturation is induced. Subsequently, mature ex vivo-generated peptide-pulsed cDC1s and cDC2s were overnight co-cultured with HA-1 TCR-transduced CD8^+^ T cells, whereupon T cell activation is evaluated using flow cytometry. **b**–**f** Representative histograms (**b**) and graphs (**c**–**f**) showing CD69 expression on T cells (**b, c**), T cell degranulation (CD107a expression)(**b,d**), and IFN-γ- (**b**, **e**) and TNF-α (**b**, **f**) production by T cells induced by short or long-peptide-pulsed (CytD treated) cDC1s. Data is shown as mean ± SEM (*n* = 3). **g** Schematic overview of antigen cross-presentation assay with patient-derived PBMCs. Mature ex vivo-generated long-peptide-pulsed cDC1s were co-cultured with alloSCT patient-derived PBMCs containing low frequencies of HA-1 specific CD8^+^ memory T cells. After 7 days of co-culture, HA-1 specific CD8^+^ T cell expansion was assessed using tetramer-based flow cytometry. **h** Representative dot plots in which numbers indicate the frequency of HA-1 specific CD8^+^ T cells. **i**–**j** Frequencies and absolute numbers of HA-1 CD8^+^ T cells of three different patients. Patient characteristics are shown in supplementary table S1. Statistical analysis was performed using unpaired T test (**b**, **c**) or repeated measures one-way ANOVA followed by Bonferroni post-hoc test (**e**–**h**). ***P < 0.001, **P < 0.01, *P < 0.05. CytD, cytochalasin D; TCR, T cell receptor; NS, non-significant; SEM, standard error of the mean

One of the hallmark features of cDC1s, compared to cDC2s and pDCs, is their superior capacity to cross-present exogenous antigens in major histocompatibility complex (MHC) class I molecules to CD8^+^ T cells [13, 14, 26–28]. Therefore, we first investigated the cross-presentation capacity of our ex vivo-generated cDC1s and cDC2s using HA-1 long-peptide in a model system with HA-1 TCR-transduced CD8^+^ T cells (Fig. 5a) [23]. Both HA-1 short- and long-peptide-pulsed ex vivo-generated cDC1s effectively induced activation of HA-1 TCR-transduced T cells, demonstrated by increased CD69 expression (Fig. 5b–c). Additionally, the cDC1-stimulated T cells acquired diverse effector functionalities, shown by increased degranulation potential, and IFN-*γ* and TNF-*α* production (Fig. 5b, d–f). Notably, titration experiments demonstrated that cDC1s also effectively (cross-)presented lower concentrations of HA-1 short- and long-peptides (Fig. S5a–b). Short- or long-HA-1 peptide only, in absence of cDC1s, did not induce T cell activation (Fig. S5c–d). To prove true antigen cross-presentation capacity of our cDC1s, cytochalasin D (CytD) was added to block actin-polymerization and prevent phagocytosis [29, 30]. CytD treatment of HA-1 short-peptide-pulsed cDC1s significantly decreased CD69 expression and IFN-γ production, but not degranulation nor TNF-α production by HA-1 TCR-transduced T cells (Fig. 5b–f). In contrast, CytD-mediated interference of long-peptide-pulsed cDC1s, completely abrogated CD69 expression, degranulation, IFN-γ- and TNF-α production by the T cells (Fig. 5b–f). CytD treatment did not negatively affect cDC1 maturation nor cell viability (Fig. S5e-f). More importantly, cDC1s also efficiently cross-presented HA-1 long-peptide and induced expansion of patient’ HA-1-specific CD8^+^ T cells ex vivo. For three patients, we found increased frequencies and absolute numbers of HA-1-specific CD8^+^ T cells (Fig. 5g–j). *Ex* vivo-generated cDC2s also efficiently induced expansion of patient-derived HA-1-specific CD8^+^ T cells in 2 out of 3 patients (Fig. S5g-h), though they were less powerful in cross-presenting HA-1 long-peptide and subsequently activating HA-1 TCR-transduced T cells as compared to cDC1s, reflected by lower expression levels of activation maker CD137 and degranulation (Fig. S5i-j). Together, these data demonstrate that our ex vivo-generated cDC1s superiorly cross-present tumor antigens and thereby competently boost the expansion of patient-derived tumor-reactive HA-1 specific CD8^+^ T cells ex vivo.

### Ex vivo-generated cDC1s are potent inducers of NK cell activation and anti-leukemia reactivity

**Fig. 6:**
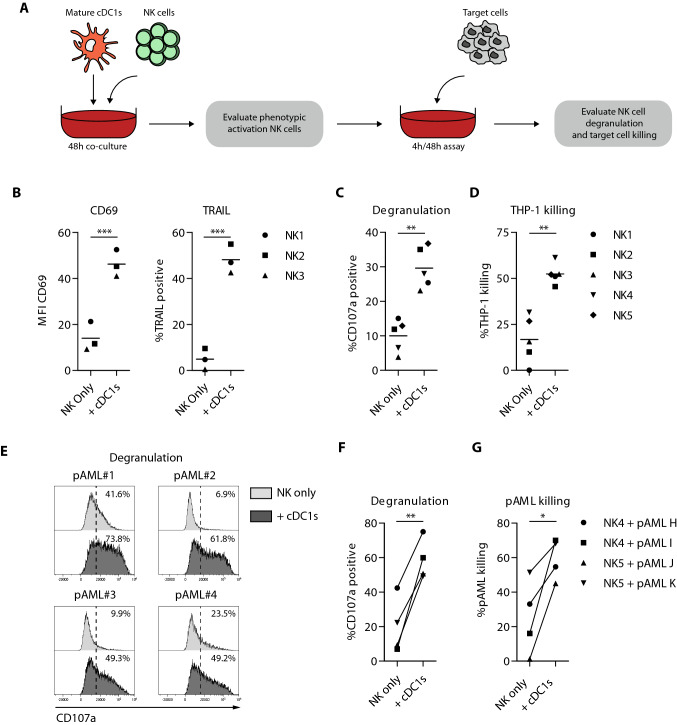
Ex vivo-generated cDC1s effectively enhance NK cell leukemia reactivity. **a** Schematic overview of NK cell activation, degranulation and killing assays. Mature ex vivo-generated cDC1s were co-cultured with NK cells (1:1) for 48 h, followed by evaluation of phenotypic NK cell activation. Additionally, 48 h cDC1-activated NK cells were cultured with the AML cell line THP-1 for 4 h or primary AML cells for 48 h, whereupon NK cell degranulation and target cell killing were evaluated. **b** MFI of CD69 and expression of TRAIL on cDC1-activated NK cells after 48 h co-culture and prior to 4/48 h degranulation and killing assay. Lines indicate mean value (*n* = 3 independent NK cell donors). **c**, **d** Degranulation by (**c**) and killing of THP-1 cells (**d**) by cDC1-activated NK cells. Lines indicate mean value (*n* = 5 independent NK cell donors). **e**–**g** Degranulation by (**e, f**) and killing of primary AML cells (**g**) by cDC1-activated NK cells. Lines indicate mean value (*n* = 4). Statistical analysis was performed using paired T test (**b**–**d**, **f** and** g**). ***p < 0.001, **p < 0.01, *P < 0.05. NK, natural killer; pAML, primary acute myeloid leukemia; MFI, median fluorescence intensity; TRAIL; TNF-related apoptosis-inducing ligand

Besides T cells, NK cells are key players in graft-versus-tumor immunity [3, 31, 32]. To assess the NK cell stimulatory capacity of our ex vivo-generated cDC1s, cDC1s were co-cultured with healthy donor-derived NK cells (Fig. 6a). Positive control data, of NK cells activated with high dose rh IL-15, are provided in Fig S6A. Interestingly, ex vivo-generated cDC1s strongly boosted NK cell activation in a dose-dependent manner, shown by increased expression of CD69 and TNF-related apoptosis-inducing ligand (TRAIL) (Fig. 6b, S6b). Furthermore, these cDC1-activated NK cells displayed dose-dependent enhanced anti-leukemic reactivity, reflected by increased degranulation and effective killing of THP-1 (Fig. S6c–d), KG1a and HL-60 AML cell lines (Fig. 6c–d; Fig. S6h–k). More importantly, cDC1-activated NK cells also responded effectively to primary AML cells, as indicated by enhanced degranulation (Fig. 6e–f) and tumor killing (Fig. 6g). Similar NK cell activating potential was observed for ex vivo-generated cDC2s (Fig. S6e–k). To conclude, these data indicate that our ex vivo-generated cDC1s potently orchestrate innate leukemic-reactive NK cell responses.

## Discussion

Although alloSCT, following induction chemotherapy, can be curative for hemato-oncology patients [1], relapse remains the major cause of treatment failure. This can amongst others be attributed to inadequate immune-mediated tumor control, illustrating the need for tailored adjuvant immunotherapy to boost and strengthen anti-tumor immunity post alloSCT. In this perspective, natural DC vaccination is highly attractive as these cells are the key orchestrators of innate and adaptive anti-tumor T and NK cell responses. Here, we describe for the first time a robust clinically applicable ex vivo culture protocol, based on GMP-grade materials and devoid of animal components, which allows simultaneous generation of pDCs, cDC2s and cDC1s from donor-derived G-CSF mobilized CD34^+^ HPCs. Notably, our protocol is amongst the first to successfully match ex vivo-generated blood DC subsets with their natural counterparts based on transcriptome, phenotype and function.

Compared with previously published CD34^+^ stem cell-based ex vivo culture protocols studies by us and others, we omitted the use of the OP9-DL1/4 murine bone marrow stromal cells, and introduced IFN-α [16–20, 27]. It has been shown that type I IFN signaling induces expression of the FLT3L receptor, which is one of the most crucial cytokines for DC development [33, 34]. We showed that addition of IFN-α resulted for the first time in effective generation of large numbers of all three DC subsets using one GMP-grade culture protocol and is a significant step forward toward clinical application of combined pDCs, cDC2 and cDC1 vaccination post alloSCT. Per input G-CSF-mobilized CD34^+^HPC we generated approximately ~ 6 cDC1s, ~ 21 cDC2s and ~ 10 pDCs, which is higher compared to the ex vivo OP9-DL1 based culture protocol of Kirkling et al. [17] using bone marrow-derived CD34^+^ HPCs (~ 4 cDC1s), but lower compared to the ex vivo OP9-DL1 based culture protocol of Balan et al. [16] using cord blood-derived CD34^+^ HPCs (~ 11 cDC1s, ~ 41 pDCs). This may be anticipated as cord blood-derived progenitor cells are well-known for their superior expansion potential compared with adult stem cells [35]. In the context of alloSCT for hematological malignancies, G-CSF mobilized CD34^+^ HPCs are the most commonly used source for transplantation. Hence, we focused our studies on development of a tailored adjuvant immunotherapeutic strategy using this HPC source. Importantly, our culture system is easily scalable. For instance, starting with 10 × 10^6^ G-CSF-mobilized CD34^+^HPCs (*i.e.,* < 3% of the G-CSF mobilized donor graft), we can generate quadruple the number of cDC2s and pDCs as compared to isolation from PB using CliniMACS as reported in clinical trials (on average cDC2s: 210 × 10^6^ ex vivo vs. 55 × 10^6^ from apheresis; pDCs: 100 × 10^6^ ex vivo vs. 23 × 10^6^ from apheresis) [11, 12]. Notably, data on clinical apheresis yield of cDC1s are not yet available. However, as cDC1 frequencies in blood are 22-fold lower than those of pDCs [36], an estimated yield of 1 × 10^6^ may be expected from apheresis compared to 63 × 10^6^ using our ex vivo culture protocol. This underscores the advantage of using our HPC-derived DC subsets for post-alloSCT vaccination purposes as compared to blood isolated DCs.

Notably, mRNA deep sequencing analyses revealed that our ex vivo-generated DC subsets highly resemble their in vivo counterparts, strongly supporting their specific identity. Expression of previously described key genes was consistent in our respective ex vivo-generated DC subsets [25, 37]. We found low *CD1C* expression in our ex vivo-generated cDC1s, which may be attributed to GM-CSF driven induction of *CD1a-c* gene expression [9, 38]. In addition to transcriptomic resemblance, we demonstrated that our ex vivo-generated DCs exhibited key functional features. In previous studies and supplemental data, we reported highly powerful induction of anti-tumor T and NK cell responses by ex vivo-generated cDC2s and pDCs [18]. Hence, we further focused on the functional characterization of the rare cDC1 subset. We demonstrated that ex vivo-generated cDC1s can potently boost innate and adaptive immunity. Most importantly, we demonstrated that they truly harbor the key phenotypic and functional properties of their in vivo counterpart. Upon TLR-mediated maturation, our ex vivo-generated cDC1s highly upregulated chemokine receptor CCR7 and co-stimulatory molecules CD80, CD83 and CD86, and released pro-inflammatory cytokines IL-12p70 and TNF-*α*, comparable with in vivo cDC1s. Additionally, they efficiently migrated toward lymph node homing chemokine CCL21 in vitro, important for proper priming of naïve T cells. We further showed that our ex vivo-generated cDC1s effectively induced priming of naïve alloreactive T cells and augmented the expansion of antigen-experienced healthy donor-derived CMV and patient-derived MiHA-specific CD8^+^ T cells. Moreover, ex vivo-generated cDC1s superiorly cross-presented long MiHA HA-1 peptide to HA-1 TCR-transduced T cells and patient-derived HA-specific CD8^+^ T cells [13, 39]. Importantly, even in more strict situations with lower peptide concentrations our cDC1s effectively activated HA-1 specific CD8^+^ T cells, with higher potency for short-peptide as compared with long-peptide as expected and according to the literature [40, 41]. Notably, cDC cross-presentation ability was completely abrogated upon CytD-mediated interference of antigen uptake, showing their true antigen cross-presentation capacity. The lower CD69 expression and IFN-γ production by T cells upon CytD treatment of short-peptide-pulsed cDC1s might be attributed to decreased phagocytosis of the HA-1 short-peptide.

Besides T cells, NK cells play an important role in graft-versus-tumor immunity [31, 42]. We demonstrated potent cDC1-mediated NK cell activation, and increased NK-mediated reactivity against THP-1 AML cells. The NK cell stimulatory properties of our ex vivo-generated cDC1s might be attributed to IL-12 and IL-15 secretion and subsequent IL-15 trans-presentation [43, 44]. In other studies, IL-12 was shown to rescue cytotoxic properties of NK cells against metastatic melanoma [45] and to be important in immunological synapse formation between DCs and NK cells [46]. Next to DC-NK cell interactions, also the NK-target cell interactions modulate the cytolytic potential of the NK cells. Here, multiple factors are implicated, including KIR-ligand mismatch between NK cells and target cells [47] the expression of activating and/or inhibitory receptors on NK cells, which might also be influenced by the DCs, and the expression of activating and/or inhibitory receptors on the target cells [48].

To induce broad and potent anti-tumor immunity in vivo, combined administration of cDC1s, cDC2s and pDCs is highly attractive, as they possess distinctive functional features. Increasing evidence shows direct and indirect cross-talk between cDCs and pDCs, as well as cDC1s and cDC2s. pDC-derived IFN-α enhances maturation, IL-12p70 production and immune stimulatory capacity of cDCs [8]. Most of the in vivo cross-talk studies have been performed in viral-infection models using mouse DCs. For example, Brewitz et al*.* [49] demonstrated that vaccinia virus Ankara-infected cDC1s recruit pDCs in a CCR5-dependent fashion to the CD8^+^ T cell priming site. These pDCs produced high levels of IFN-α, driving cDC maturation and antigen-presentation ability, which led to robust anti-viral CD8^+^ T cell responses. Another study reported that combined cDC2 and pDC administration resulted in increased frequencies and IFN-γ production of tumor-reactive T cells in an OVA-specific tumor model [50]. Notably, robust activation of CD8^+^ T cell responses requires both cDC1s and cDC2s in viral and tumor models [51, 52]. In future studies, we will assess the potency of our ex vivo-generated cDC1s, cDC2 and pDCs to orchestrate in vivo tumor-reactive T and NK cell responses and define the optimal vaccination strategy and vaccine composition. Furthermore, as the purity of the DC subset product is ~ 31% at the end of the culture, we will then also further characterize the ‘non-DCs’ and assess their potential impact on cDC and pDC functionality. Depending on these follow-up studies, potential optimizations include further tweaking of the culture protocol to increase the DC purity and/or enrich DC subsets for vaccination purposes.

In conclusion, we have developed a novel clinically applicable culture protocol to simultaneously and robustly generate large numbers of ex vivo pDC, cDC2 and the rare cDC1 subsets which strongly resemble their in vivo counterparts. Upon maturation, ex vivo-generated cDC1s acquired key subset-specific phenotypic and functional properties, facilitating effective activation of tumor-reactive T and NK cell responses. Together, this makes our ex vivo-generated cDC1s, beside cDC2s and pDCs, powerful means to further boost and maintain innate and adaptive anti-tumor immunity in vivo. This holds strong potential to establish long-lasting tumor control, and improve relapse-free and overall survival of hemato-oncology patients post alloSCT.

## Supplementary Information

Below is the link to the electronic supplementary material.

Supplementary file1 (PDF 62500 KB)

## Data Availability

Data is available on reasonable request.
