# Supplementary material for: Clinically applicable CD34+-derived blood dendritic cell subsets exhibit key subset-specific features and potently boost anti-tumor T and NK cell responses
Source: Cancer Immunol Immunother. 2021 Apr 1;70(11):3167–81. doi: 10.1007/s00262-021-02899-3 (PMC8505305; doi:10.1007/s00262-021-02899-3)
Supplement: Supplementary file 1 — Supplementary file1 (PDF 62500 KB) [file 262_2021_2899_MOESM1_ESM.pdf]

**Supplementary Appendix**

**Supplementary tables**

Supplementary Table 1. Patient characteristics

Supplementary Table 2. Overview of antibodies in flow cytometry analyses.

**Supplemental Figures**

Figure S1. Gating strategy and phenotype pf *ex vivo*-generated cDC21s, cDC2s and pDCs.

Figure S2. Sorting strategy *ex vivo*-generated and *in vivo* DC subsets.

Figure S3. *Ex vivo*-generated cDC2s and pDCs possess phenotypic and functional resemblance with their *in vivo* counterparts

Figure S4. *Ex vivo*-generated cDC2s and/or cDC1s possess LN homing capacity, efficiently induce alloreactive T cell proliferation and boost expansion of tumor-reactive T cells.

Figure S5. Short and long-peptide titration, control conditions in antigen cross-presentation assays and antigen cross-presentation by ex vivo-generated cDC2s.

Figure S6. *Ex vivo*-generated cDC1s and cDC2s enhance NK cell-mediated anti-leukemic reactivity.

**Supplemental tables**

**Supplementary Table 1. Patient characteristics**

| Patient | Disease | Sample time point | Sample type | Extra information | MiHA | % MiHA-specific T cells day 0 |
|---|---|---|---|---|---|---|
| **A** | Multiple myeloma | 22 mo post alloSCT | PB | DLI: 6 mo post alloSCT | HA-1 | 0.06 |
| **B** | Myeloid dysplastic syndrome | 9 mo post alloSCT | PB | - | HA-1 | 0.25 |
| **C** | Acute myeloid leukemia | 12 mo post alloSCT | PB | - | HA-1 | 0.02 |
| **D** | Myeloid dysplastic syndrome | 9 mo post alloSCT | PB | DLI: 4 mo post alloSCT | LRH-1 | 0.05 |
| **E** | Acute myeloid leukemia | 36 mo post alloSCT | PB | - | LRH-1 | 0.02 |
| **F** | Acute myeloid leukemia | 76 mo post alloSCT | PB | DLI: 13 and 36 mo post alloSCT | LRH-1 | 0.03 |
| **G** | Myeloid dysplastic syndrome | 3 mon post alloSCT | PB | - | HA-1 | < 0.01 |
| **H** | Acute myeloid leukemia M0 | Diagnosis | BM | - | - | - |
| **I** | Acute myeloid leukemia M1 | Diagnosis | BM | - | - | - |
| **J** | Acute myeloid leukemia M2 | Diagnosis | BM | - | - | - |
| **K** | Acute myeloid leukemia M0 | Diagnosis | BM | - | - | - |

Mo, months; DLI, donor lymphocyte infusion; alloSCT, allogeneic stem cell transplantation; MiHA; Minor histocompatibility antigen

**Supplementary Table 2. Overview of used antibodies in flow cytometry analyses.**

| Specificity | Fluorochrome | Clone | Isotype | Company | Cat# |
|---|---|---|---|---|---|
| **HPC Purity** | | | | | |
| CD34 | PE-Cy7 | 581.0 | IgG1κ | Biolegend | A21691 |
| CD45 | ECD | J.33 | IgG1κ | Beckman Coulter | A007784 |
| **Phenotype and sort *ex vivo* generated DCs** | | | | | |
| CD1c | APC-Cy7 | L161 | IgG1κ | Biolegend | 331520 |
| CD14 | PE-Cy7 | HCD14 | IgG1κ | Biolegend | 325618 |
| CD123 | BV510 | 6H6 | IgG1κ | Biolegend | 306022 |
| CD141 | APC | M80 | IgG1κ | Biolegend | 344106 |
| CD303 | FITC | 201A | IgG2aκ | Biolegend | 353208 |
| CD370/CLEC9A | PE | 8F9 | IgG2a | Biolegend | 353804 |
| HLA-DR | ECD | Immu359 | IgG1κ | Beckman Coulter | IM3636 |
| **Phenotype and sort *in vivo* DCs** | | | | | |
| CD1c | PE-Cy7 | L161 | IgG1κ | Biolegend | 331516 |
| CD11c | AF700 | Bu15 | IgG1κ | Biolegend | 337220 |
| CD14 | BV785 | M5F2 | IgG2aκ | Biolegend | 301841 |
| CD19 | PE | HIB19 | IgG1κ | eBioscience | 12-0193-82 |
| CD141 | APC | M80 | IgG1κ | Biolegend | 344106 |
| CD303 | FITC | 201A | IgG2aκ | Biolegend | 353208 |
| HLA-DR | APC Cy7 | G45-6 | IgG2aκ | BD Bioscience | 335976 |
| **DC maturation assays** | | | | | |
| CD80 | PE-Cy7 | L307.4 | IgG1κ | BD Bioscience | 561135 |
| CD83 | PE | HB15e | IgG1κ | Biolegend | 305308 |
| CD86 | AF488 | IT2.2 | IgG2bκ | Biolegend | 305414 |
| CCR7 | FITC | G043H7 | IgG2aκ | Biolegend | 353216 |
| **Allogeneic T cell proliferation assays** | | | | | |
| CD3 | PE-Cy7 | UCHT1 | IgG1κ | Biolegend | 300420 |
| CD8 | AF700 | RPA-T8 | IgG2aκ | Invitrogen | MCHD0829 |
| **Antigen-specific T cell expansion assays** | | | | | |
| CD3 | PE-Cy7 | UCHT1 | IgG1κ | Biolegend | 300420 |
| CD8 | AF700 | RPA-T8 | IgG2aκ | Invitrogen | MCHD0829 |
| Tetramer | PE | - | - | Custom | |
| Tetramer | APC | - | - | Custom | |
| **Cross-presentation assays** | | | | | |
| CD8 | AF700 | RPA-T8 | IgG2aκ | Invitrogen | MCHD0829 |

| | | | | | |
|---|---|---|---|---|---|
| CD69 | BV421 | FN50 | IgG1κ | Biolegend | 310930 |
| CD107a | PE Cy7 | H4A3 | IgG1κ | Biolegend | 328618 |
| CD137 | APC | 4B4-1 | IgG1κ | BD Bioscience | 550890 |
| IFN-γ (intracellular) | FITC | B27 | IgG1κ | BD Bioscience | 554700 |
| TNF-α (intracellular) | PE | MAb11 | IgG1κ | BD Bioscience | 554513 |
| **NK cell assays** | | | | | |
| CD69 | FITC | FN50 | IgG1κ | Biolegend | 310904 |
| CD107a | PE-Cy7 | H4A3 | IgG1κ | Biolegend | 328618 |
| TRAIL/CD253 | APC | RIK-2 | IgG1κ | Biolegend | 308210 |
| **Viability Dyes** | | | | | |
| - | Sytox Blue | - | - | Invitrogen | S34857 |
| - | 7-AAD | - | - | Sigma Aldrich | A9400 |
| - | eFluor780 | - | - | Thermo Fisher | 65-0865-14 |
| - | DAPI | - | - | Sigma Aldrich | D9532-50MG |

Data acquisition and analyses were performed using Gallios or FC500 flow cytometers and Kaluza version 2.1 software (all Beckman Coulter).

**Abbreviations**

CD: Cluster of differentiation

HPC: Hematopoietic progenitor cell

PE: Phycoerythrin

Cy: Cyanine

ECD: Electron coupled dye (PE-Texas red)

APC: Allophycocyanin

BV: Brilliant violet

FITC: Fluorescein

AF: Alexa fluor

## Supplemental Figure 1

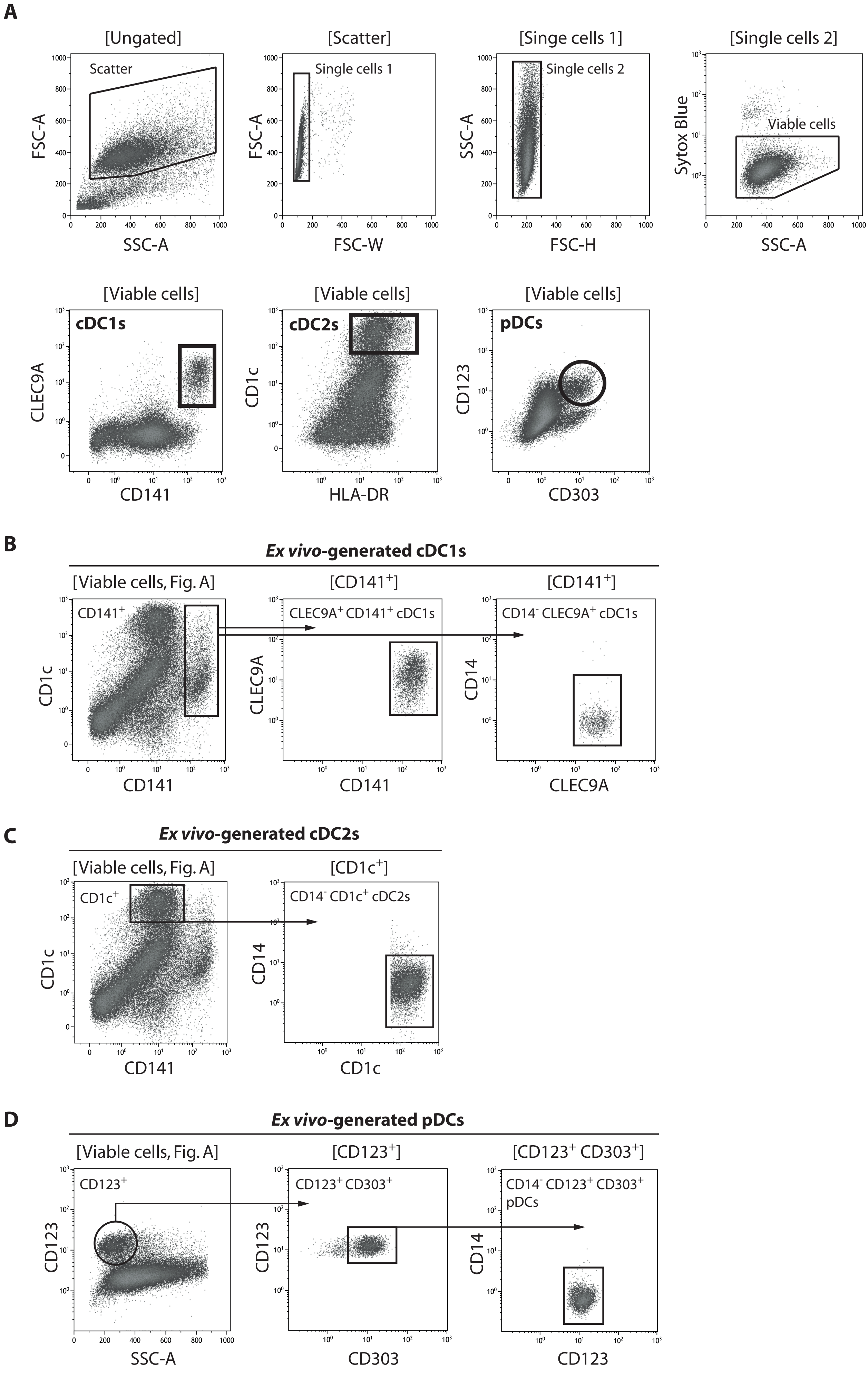

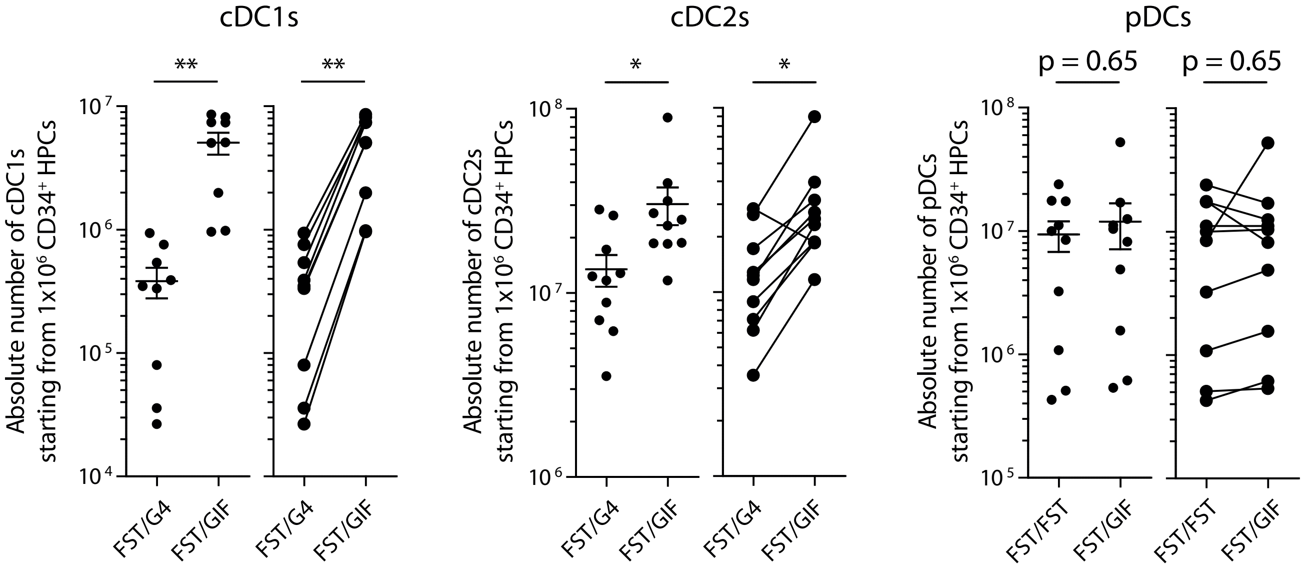


**Supplementary Figure 1. Gating strategy and phenotype of *ex vivo*-generated cDC1s, cDC2s and pDCs.** (A) Dot plots of a representative FST/GIF *ex vivo* HPC-DC culture showing gating strategy of HPC-derived DC subsets within total cultured cells. Cells are gated based on forward- and side scatter, doublets (FCS-A/SSC-A versus FCS-W/FCS-H) and dead cells (sytox blue positive) were excluded, followed by gating on the particular HPC-derived DC subsets using CLEC9A and CD141 for cDC1s, CD1c and HLA-DR for cDC2s and CD123 and CD303 for pDCs. (B-D) All *ex vivo*-generated DC subsets are $CD14^-$ (E) Absolute number of *ex vivo*-generated cDC1s, cDC2s and pDCs in the FST/GIF culture compared with previously reported FST/G4 and FST/FST culture protocols (18).

## Supplemental Figure 2

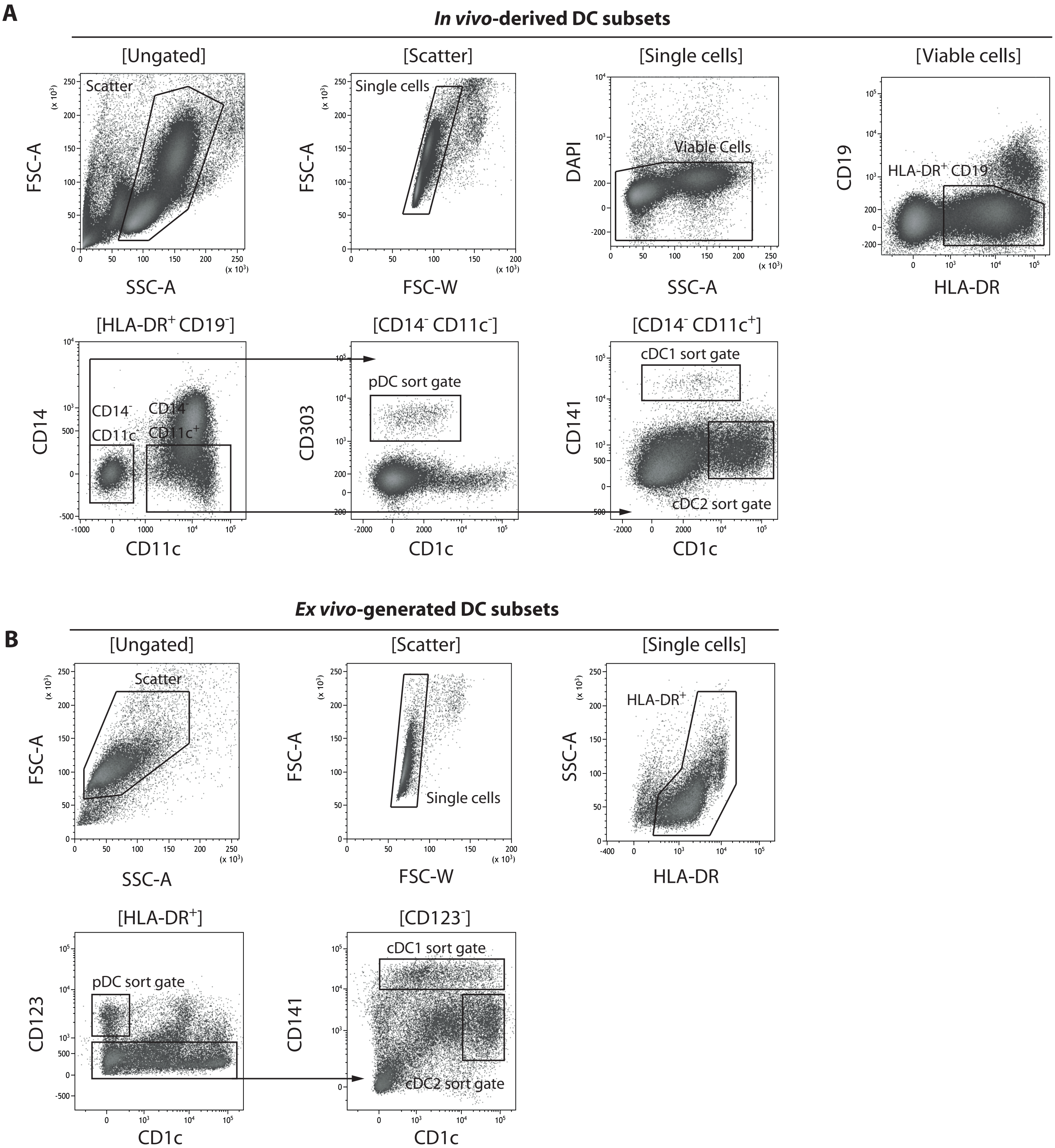


**Supplementary Figure 2. Sort strategy *ex vivo*-generated- and *in vivo* DC subsets.** (A-B) Dot plots of representative sorting strategy of *in vivo*-derived- (A) and *ex vivo*-generated (B) DC subsets.

**Supplemental Figure 3**

**A**

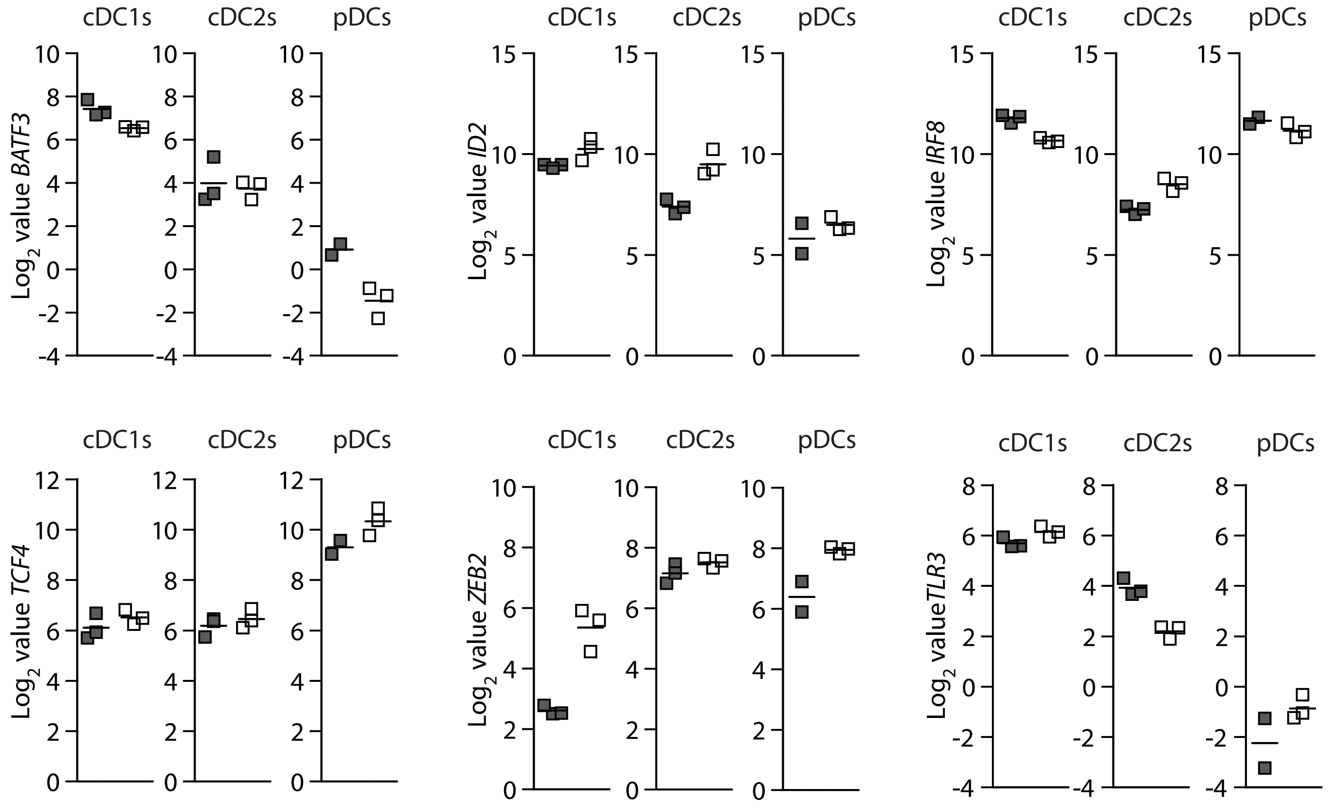


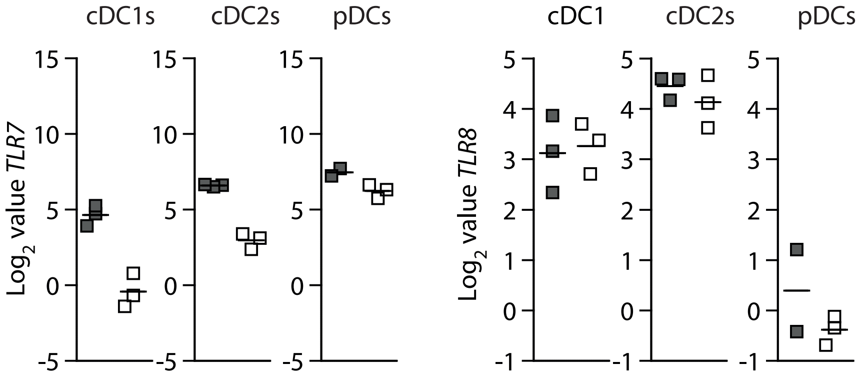


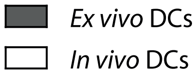


| | cDC1s | cDC2s | pDCs |
|---|---|---|---|
| *BATF3* | High | Med | Low |
| *ID2* | High | High | Low |
| *IRF8* | High | Med | High |
| *TCF4* | Low | Low | High |
| *ZEB2* | Low | High | High |
| *TLR3* | High | Med | Low |
| *TLR7* | Low | Low | High |
| *TLR8* | High | High | Low |

Adapted from Collin *et al.*[9]

**B** **C**

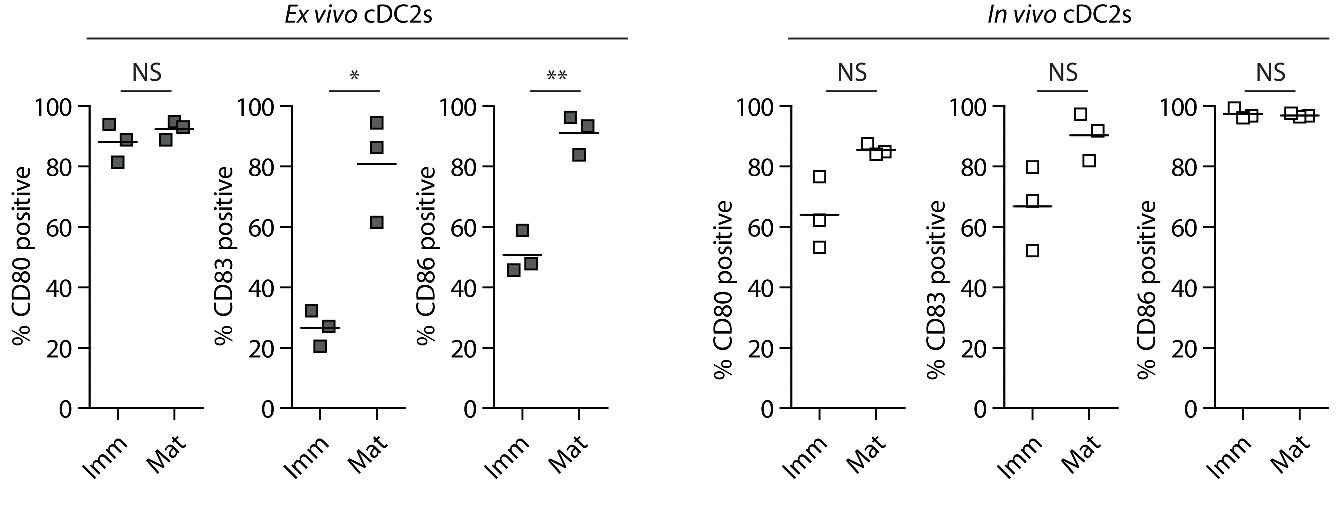

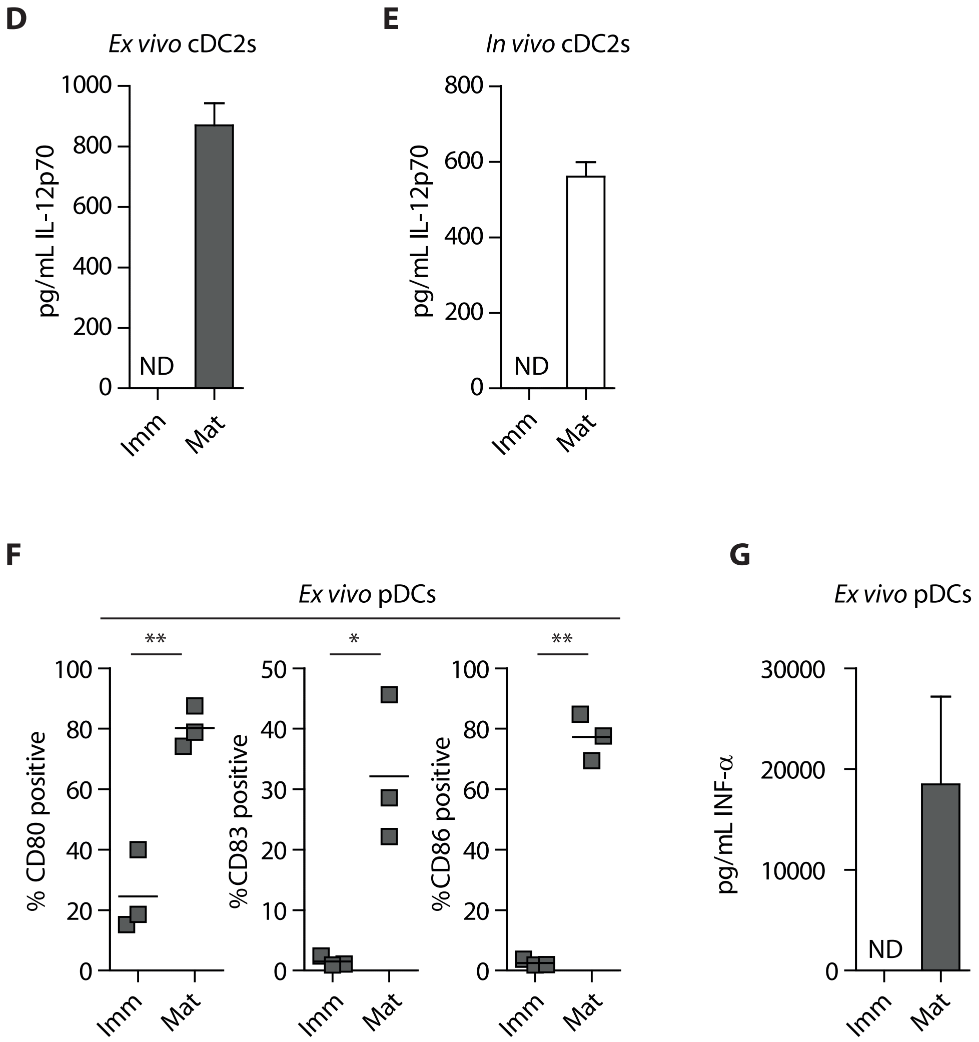


**Supplementary Figure 3.** ***Ex vivo*-generated cDC2 and pDCs possess phenotypic and functional resemblance with their *in vivo* counterpart.** (A) Expression of cDC1, cDC2 and pDC-related transcription factors and TLRs. (B-C) Expression of co-stimulatory molecules CD80, CD83 and CD86 on immature and mature *ex vivo*-generated (B) and *in vivo* cDC2s (C). (D-E) Release of pro-inflammatory cytokine IL12p70 by immature and mature *ex vivo*-generated (D) and *in vivo* cDC2s (E). (F-G) Expression of co-stimulatory molecules CD80, CD83 and CD86 on immature and mature *ex vivo*-generated pDCs. (G) Release of pro-inflammatory cytokine IFN-α by immature and mature *ex vivo*-generated pDCs. Data is shown as mean ± SEM (n=3). Statistical analysis was performed using paired T-test (B). TLR, toll like receptor. *** P < 0.001, ** P < 0.01. ND, non-detectable; SEM, standard error of the mean.

## Supplemental Figure 4

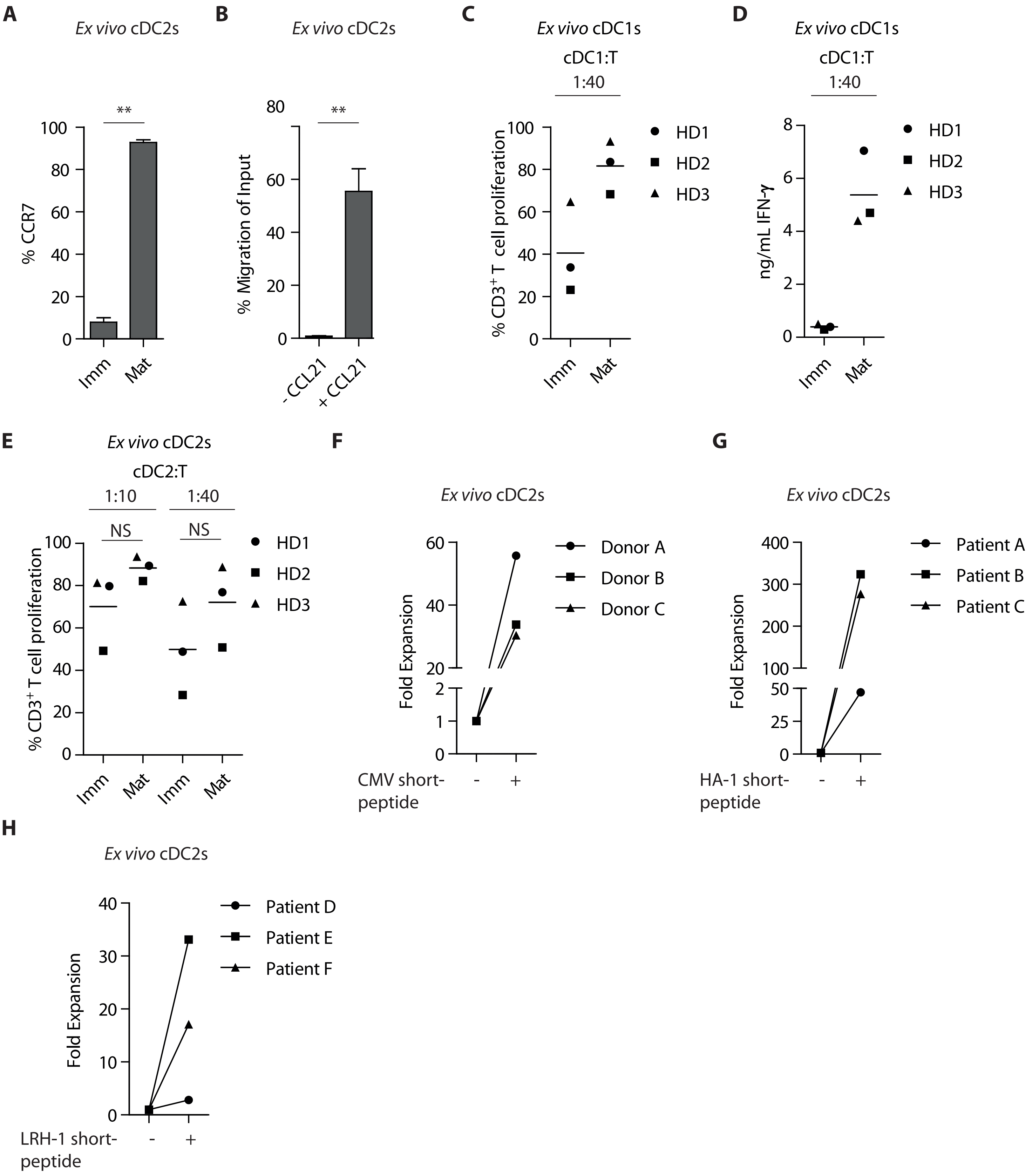


**Supplementary Figure 4. *Ex vivo*-generated cDC2s and/or cDC1s possess LN homing capacity, efficiently induce alloreactive T cell proliferation and boost expansion of tumor-reactive T cells.** (A) Expression of LN homing chemokine receptor CCR7 on immature and mature *ex vivo*-generated cDC2s. Data is shown as mean ± SEM (n=3). (B) Percentage of *ex vivo*-generated cDC2 migration to LN homing chemokine CCL21 (250 ng/mL). Data is shown as mean ± SEM (n=3). (C, E) Percentage of allogeneic T cell proliferation at day 5 of culture induced by *ex vivo*-generated cDC1s (C) or *ex vivo*-generated cDC2s (E) using cDC:T cell ratio of 1:10 or 1:40 respectively. Lines indicate mean value (n=3). (D) Release of IFN-γ upon 5 days T cell stimulation using *ex vivo*-generated cDC1s in alloMLRs using a cDC1:T cell ratio of 1:40. Line indicate mean value (n=3). (F-H) Fold expansion of CMV, HA-1 -and LRH-1 specific $CD8^+$ T cell induced by short-peptide-pulsed *ex vivo*-generated cDC2s. Statistical analyses was performed using an unpaired T test (A, C-D) or paired T test (B) LN, lymph node; CCR7, C-C chemokine receptor 7; CCL21, C-C motif chemokine ligand 21; AlloMLRs, allogeneic mixed leukocyte reactions; SEM, standard error of the mean. ** P<0.05.

## Supplemental Figure 5

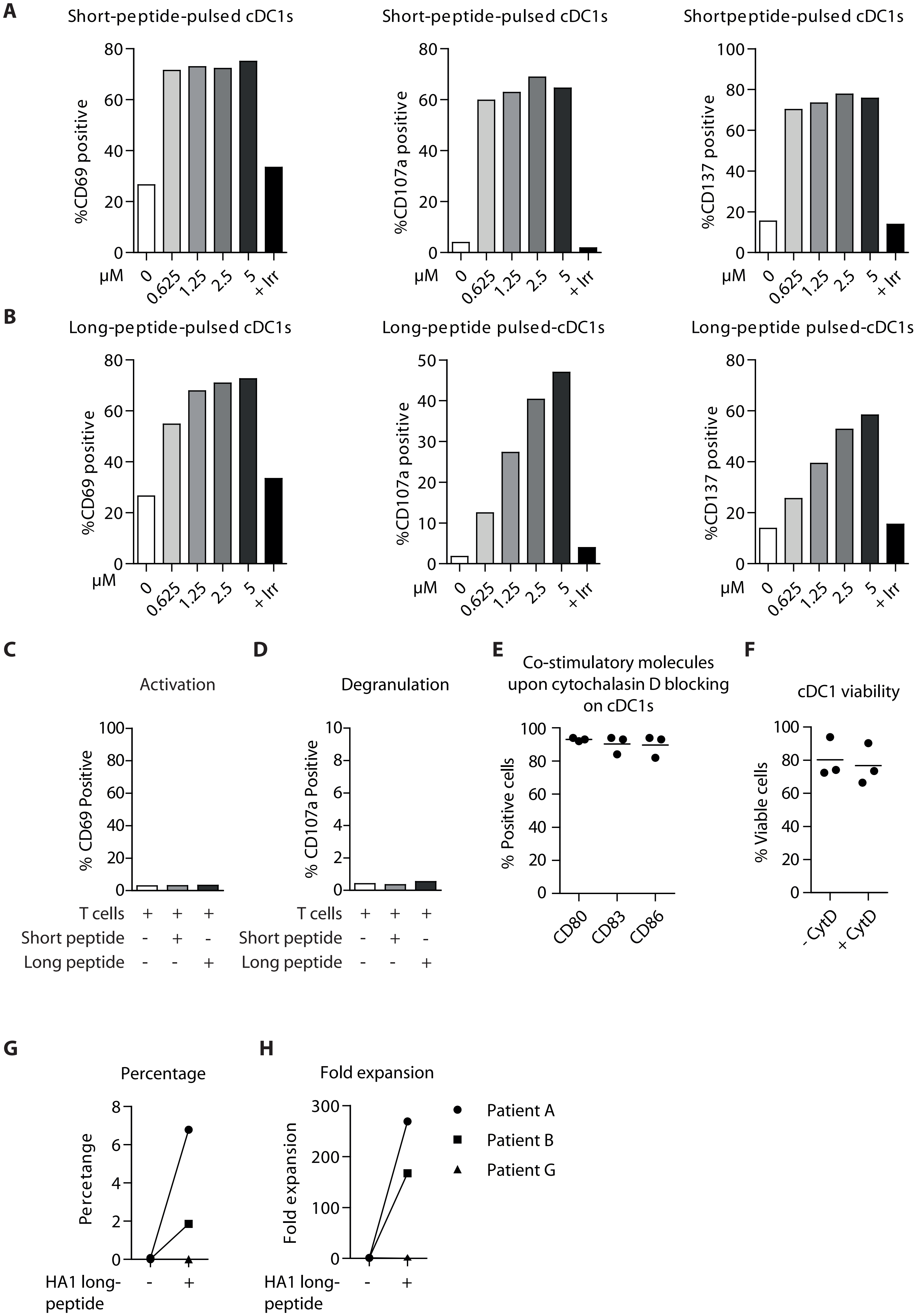

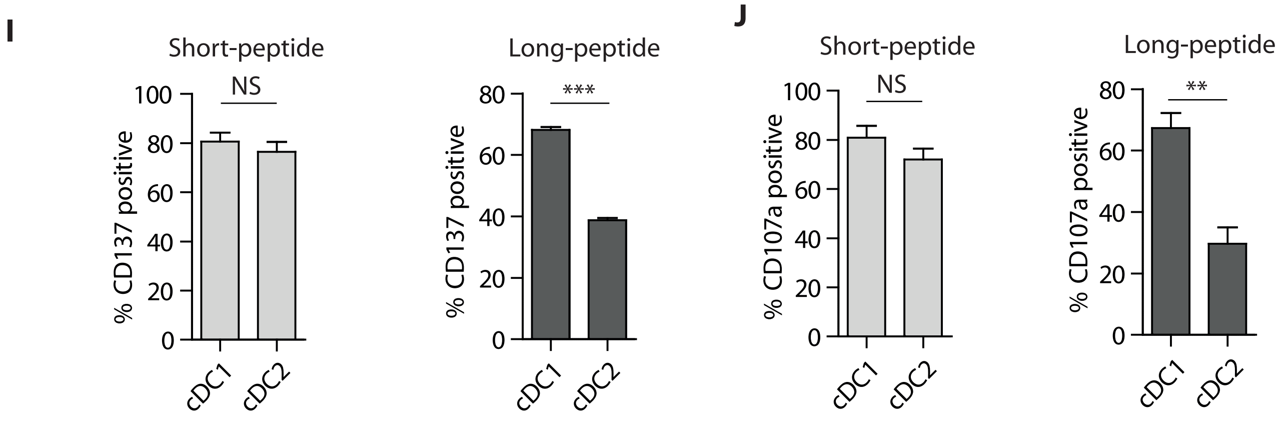


**Supplementary Figure 5. Short- and long-peptide titration, control conditions in antigen cross-presentation assays and antigen cross-presentation by *ex vivo*-generated cDC2s.** (A-B) Activation shown by expression of CD69, CD107a and CD137 of HA-1 T cell receptor (TCR) transduced T cells by cDC1s pulsed with different concentration of short- and long HA-1 peptide. (C-D) Expression of activation marker CD69 (A) and T cell degranulation (B) by HA-1 TCR-transduced T cells in the absence of *ex vivo*-generated cDC1s or presence of HA-1 short- or HA-1 long-peptide only. (E-F) Expression of co-stimulatory molecules CD80, CD83 and CD86 on *ex vivo*-generated cDC1s treated with CytD. Lines indicate mean value (n=3). (D) Viability of *ex vivo*-generated cDC1s treated with/without CytD. Lines incubate mean value (n=3). (G-H) Percentage and fold expansion of patient-derived HA-1-specific T cells induced by long-peptide pulsed cDC2s. (I-J) Graphs showing CD137 expression and degranulation (CD107a expression) by HA-1-transduced TCR T cells induced by short -or long-peptide-pulsed cDC1s and cDC2s. Irr, irrelevant peptide; CytD, cytochalasin D.

**Supplemental Figure 6**

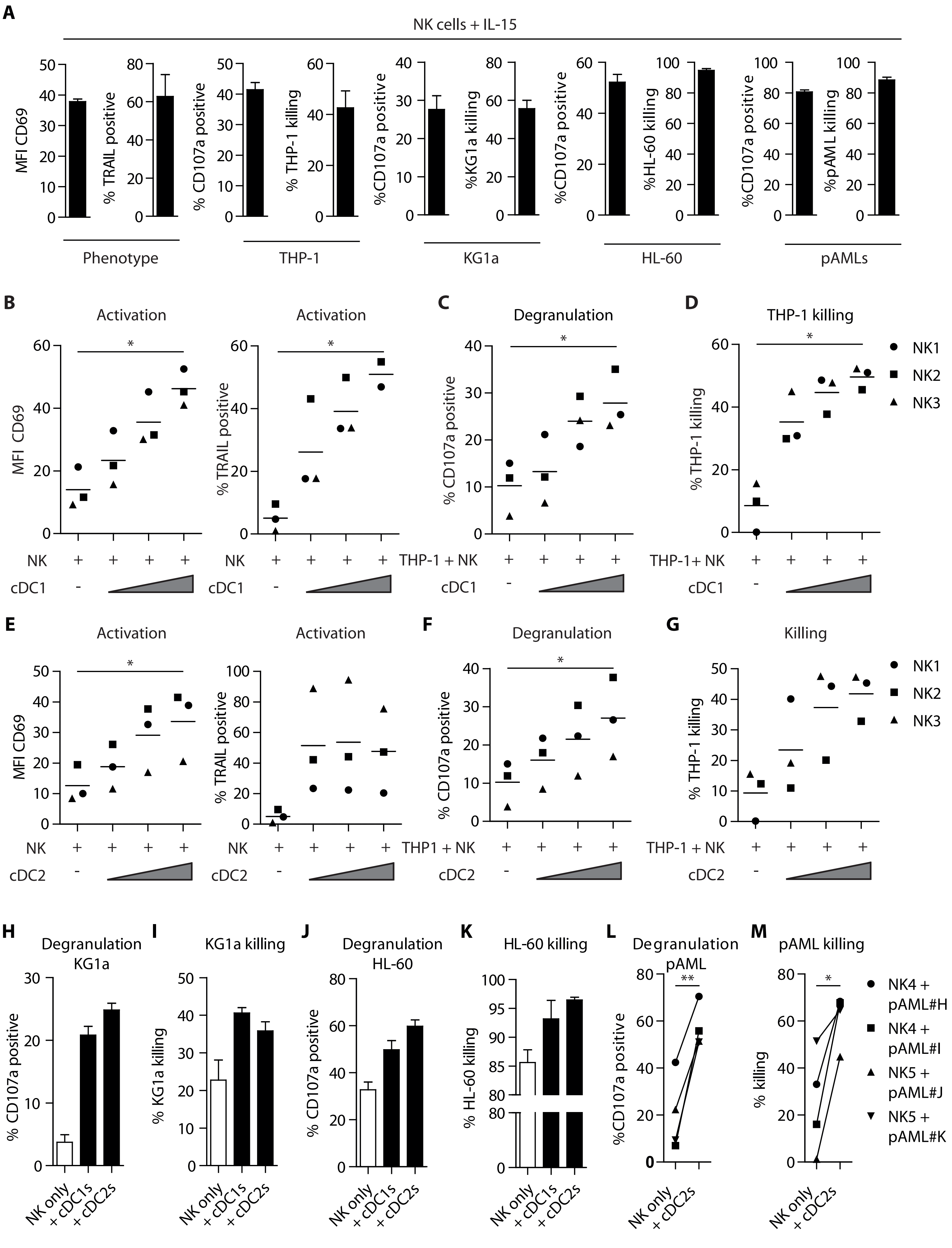

**Supplementary Figure 6. *Ex vivo*-generated cDC1s and cDC2s enhance NK cell leukemia-reactivity.** (A) Expression of CD69 and TRAIL, degranulation and tumor cell killing by IL-15-activated NK cell. Data is shown as mean ± SEM (n=3). (B, E) MFI of activation marker CD69 and percentage TRAIL expression on cDC1 and cDC2-activated NK cells after 48 hours co-cultured (cDC:NK cell ratios of 0.02:1, 0.2:5 and 1:1 respectively). Lines indicate mean value (n=3). (C, D, F, G) Degranulation (C, F) and THP-1 killing (D, G) by cDC1 and cDC2-activated NK cells 4 hours stimulated with THP-1 cells (NK cell:THP-1 cells ratio of 2:1). Lines indicate mean value. (H-K) Degranulation (H, J) and killing (I,K) by cDC1 and cDC2-activated NK cells 4 hours stimulated with KG1a (H-I) or HL-60 (J-K). (L, M) Degranulation by (L) and killing of pAML (M) by cDC2-activated NK cells 48 hours stimulated with primary AML cells. Statistical analyses was performed using Friedman test followed by Dunns post-hoc test (B, C, D, E and F) or paired T-test (L, M). * $P < 0.05$. NK, natural killer; TRAIL, TNF-related apoptosis-inducing ligand; pAML, primary acute myeloid leukemia; MFI, median fluorescence intensity.
